# A descriptive study of pain treatment and its follow-up in primary care of elderly patients after orthopaedic care

**DOI:** 10.1186/s40780-020-00166-8

**Published:** 2020-05-04

**Authors:** Gabriella Caleres, Patrik Midlöv, Åsa Bondesson, Sara Modig

**Affiliations:** 1 Department of Clinical Sciences in Malmö/Center for Primary Health Care Research, Lund University, Box 50332, 202 13 Malmö, Sweden; 2 Department of Medicines Management and Informatics in Skåne County, Malmö, Sweden

**Keywords:** Aged, Primary health care, Pain management, Analgesics, Opioids, Acetaminophen

## Abstract

**Background:**

Pain treatment post orthopaedic care in the elderly is complicated and requires careful follow-up. Current guidelines state all patients prescribed opioids should have a plan for gradual reduction, with the treatment progressively reduced and ended if any pain remains after more than three months. How this works in primary care remains to be explored.

The aim was to describe pain treatment and its follow-up in primary care of elderly patients after orthopaedic care.

**Methods:**

In this descriptive study, medical case histories were collected for patients ≥ 75 years, which were enrolled at two rural primary care units in southern Sweden, and were discharged from orthopaedic care. Pain medication follow-up plans were noted, as well as current pain medication at discharge as well as two, six and twelve weeks later.

**Results:**

We included a total of 49 community-dwelling patients with medication aid from nurses in municipality care and nursing home residents, ≥ 75 years, discharged from orthopaedic care. The proportion of patients prescribed paracetamol increased from 28/49 (57%) prior to admission, to 38/44 (82%) after 12 weeks. The proportion of patients prescribed opioids increased from 5/49 (10%) to 18/44 (41%). Primary care pain medication follow-up plans were noted for 16/49 patients (33%).

**Conclusions:**

Many patients still used pain medication 12 weeks after discharge, and follow-up plans were quite uncommon, which may reflect upon lacking follow-up of these patients in primary care.

## Introduction

Pain treatment in elderly patients is complicated due to age-associated physiologic, pharmacokinetic and pharmacodynamic changes as well as comorbid conditions and the tendency to have polypharmacy [1–4], notably for patients aged 75 years or older [1]. Accordingly, the safety and efficacy of the treatment may be affected, which increases the risk of adverse side effects [1–3]. Careful monitoring, especially early in treatment, can reduce this risk. It is advised to ‘start low and go slow’, i.e. carefully increasing to an individual optimal dose during close follow-up and evaluation of effect and side effects [1–6]. Time for follow-up should be documented and the treatment should also be regularly revised [1].

Mild to moderate nociceptive pain in elderly patients should primarily be treated with paracetamol according to international and national recommendations [2, 4, 7, 8]. Paracetamol is included in the World health organization’s list of essential medicines of “the most efficacious, safe and cost-effective medicines for priority conditions” [9], although both the safety and efficacy of its use is disputed [10, 11]. Analgesics such as tramadol and codeine are inappropriate for elderly due to high risk of serious side-effects, such as nausea, drowsiness and constipation, as well as drug interactions and unsure effects due to variances in metabolism [1, 2, 7]. Nonsteroidal anti-inflammatory drugs NSAIDs commonly cause side-effects and are not recommended for elderly individuals other than in reduced dosage for short periods of time for inflammatory pain [1–5]. When further pain treatment is needed due to moderate to severe persistent nociceptive pain, strong opioids may be added; an effective and well-tolerated treatment if possible side-effects are closely monitored such as obstipation, nausea, sedation and cognitive impairment [2, 3, 12, 13] as well as fall-related adverse events [14]. It is also of importance to assess the risk of opioid misuse and addiction, although commonly rather associated with young age in addition to psychiatric disorders and previous substance abuse [15]. When pain remains beyond 3 months, i.e. longer than the common healing time, it may be described as persistent or chronic and thus require a different treatment approach [7, 16, 17].

However, since drug related problems are a common cause of hospitalisation in elderly patients [18], the increased risk of side effects among this patient group may lead to under-treatment of pain, thus risking negative effects on function and quality of life [5]. Such under-treatment is commonly noted for both community-living [19] and nursing home residents [20] as well as in patients with cognitive deficits [3]. Accordingly, managing fracture-related pain in elderly patients is challenging [21]. Lack of efficient analgesia post operatively in elderly patients is linked to worse outcome [4]. Patients with cognitive deficits are even more under-treated post-operatively [22, 23].

But polypharmacy is also common among elderly patients [1], especially among nursing home residents with multi-dose drug dispensing (MDD; machine-dispensed disposable sachets in which medications are packaged according to the time of administration) [24]. MDD use is associated with a higher number of drugs and with a lower quality of drug therapy [24–27], partly due to its association with fewer changes in drug treatment, as noted in a study of elderly patients with hip fracture 6 months after discharge [28]. Such infrequent reconsideration of drug treatment including analgesics may contribute to the high prevalence of suboptimal treatment noted for MDD patients [26, 29], whose risk for overtreatment is even higher than for under-treatment [29]. Hence, persistence of drug treatment without clear indication is also a matter of concern.

Pharmacological pain management in the elderly is complicated, even more so for patients with cognitive deficits. Adequate treatment with recommended drugs requires careful follow-up, including pain assessment based on patient self-report or observation of pain behaviours [16], to minimize risk of side effects or of persistent treatment without clear indication. Current regional guidelines state that post-operative pain should be treated with paracetamol and slow-release opioids for regular and fast-acting opioids for as-needed use [7], in line with international treatment recommendations for nociceptive pain in elderly patients [2, 3]. All patients discharged with opioid treatment should have a plan for gradual reduction, and if any pain remains after more than 4 weeks, a renewed pain assessment should be performed before further prescribing of slow-release opioids [7]. If any post-operative pain remains after more than 3 months, opioid treatment should be gradually reduced and ended [7]. How this works in primary care where these patients are mainly cared for remains to be explored.

We aimed to examine pain treatment and its follow-up in primary care of elderly patients after orthopaedic care, by describing actual treatment at certain points in time from discharge until 3 months later as well as the presence of any follow-up plans regarding this treatment.

## Methods

### Setting

The study was conducted at two rural public primary care units in Skåne county in southern Sweden where **1.3 million** (13%) of the Swedish population lives [30]. This region has ten hospitals of varying sizes and just over 150 primary care units. The primary care units are generally served by the hospitals according to geographical proximity.

In this region, the hospitals and the primary care units have separate electronic medical records. Information transfer from hospital to primary care occurs by a medical case history, as well as a discharge summary which is also given to the patient and nurses in municipality care. There are no current available directives for pain assessment of community-dwelling patients with medication aid from nurses in municipality care or nursing home residents. In Sweden, such patients commonly have many chronic diseases as wells as polypharmacy [31]. Nursing homes are provided for patients with great care needs around the clock. Almost 4% of Swedes aged 65 years or older live in nursing homes, and the median age when moving in is 86 years [31].

### Selection of study subjects

**Fig. 1:**
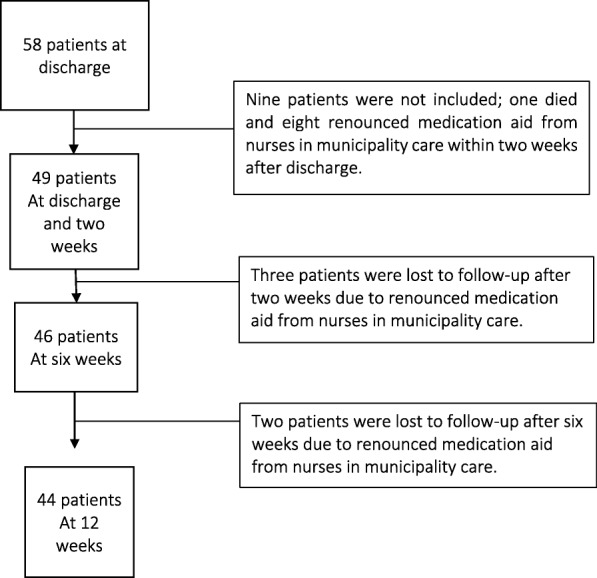
Inclusion flow chart for patients discharged from orthopaedic care to primary care

Patients were included from March 2014 until October 2017. Inclusion criteria were community-dwelling patients with medication aid from nurses in municipality care and nursing home residents (short-term or permanent), aged 75 years or older, discharged from in-patient orthopaedic care (Fig. 1).

Exclusion criteria were patient who died or renounced medical aid from nurses in municipality care within 2 weeks after discharge.

### Study design

In this descriptive study, primary care medical secretaries collected medical case histories upon arrival by post to the two primary care units, for patients aged 75 years or older that had been discharged from in-patient orthopaedic care, i.e. any hospital orthopaedic department. Whether the inclusion criteria were met was assessed by one of the authors (GC) by examining the electronic medical records and communicating with nurses in municipality care at regular weekly meetings with primary care physicians founded to enable collaboration on shared patients, or by fax (according to usual practice). By means of communicating with the nurses and examining medication lists and patient chart entries in the electronic medical records including the medical case histories and discharge summaries, as well as the medication lists in the MDD system, information on current pain medication prior to admission, at discharge as well as two, six and 12 weeks later was obtained. Residence status at discharge as well as whether the patients suffered from dementia was noted, defined as a diagnosis of a major neurocognitive disorder according to *DSM–5* in the electronic medical records. Discharge planning regarding pain medication follow-up was sought in hospital discharge documents including prescriptions, and defined as a clear instruction on how to gradually reduce and discontinue the opioid treatment. The presence or absence of referrals and discharge summaries were noted. Primary care pain medication follow-up plans in the form of patient chart entries clearly describing follow-up of the pain treatment were sought in the primary care electronic medical record. Our focus was on paracetamol and strong opioid use as these drugs form the basis of treatment of nociceptive pain in the elderly. Paracetamol was noted as either regular *or* as-needed use. The use of slow-release and/or fast-acting strong opioids were noted as regular and/or as-needed use. Regular meant being used continuously in a scheduled dosage such as once or twice daily. As-needed use meant taking the medicine according to need i.e. Pro Re Nata. Other analgesic substances such as tramadol, amitriptyline, pregabalin, prednisolone, gabapentin or paracetamol with codeine were described as ‘other pain medications’ as neither of these are considered first line treatment of nociceptive pain in elderly patients [2, 7].

### Statistical analysis

IBM SPSS Statistics 24.0 was used [32]. Descriptive statistics was used to describe the data. Categorical variables were compared using chi2 test unless for expected cell counts less than five when Fisher’s Exact Test was used. To detect any change in proportion for nominal paired data, the non-parametric McNemar test was used. A sample of 40 patients was estimated to give enough data for descriptive analyses. The level of statistical significance was set to *p* < 0.05.

### Ethical considerations

The regional ethical committee at Lund University decided there was no need for an ethics review (reference number 2015/171), but gave an advisory opinion stating that no ethical issues regarding the medical record review was seen in this study. An approval for medical record reviewing was obtained from the head of the primary care units.

## Results

A total of 49 patients were included, of which five patients were lost to follow-up at 12 weeks (Fig. 1). The average age for all patients was 86.4 years (SD 6,2 years). A total of 34 out of 49 patients (69%) were women, and 31 patients (63%) were nursing home residents. A total of 15 patients (31%) suffered from dementia, of which 12 were nursing home residents. Hip fracture was the most commonly noted diagnosis for the hospital stay (36/49, 74%).

Referrals (i.e. separate from discharge summaries) from hospital to primary care were noted for 18 out of 49 patients (37%) in total. Pain medication was the main reason for referral in two cases, of which one was received after the patient had revisited the emergency room due to severe pain after discontinued opioid treatment. Nine patients had a hospital discharge plan for gradual reduction of the opioid treatment within 3 weeks (or less) from hospital discharge. A discharge summary with medication report and medication list was noted for 19 out of 49 patients (39%). Patient chart entries regarding pain medication follow-up (i.e. primary care pain medication follow-up plans) were noted in the primary care electronic medical records for 16 out of 49 patients (33%) within 1 week from discharge. A patient chart entry regarding pain, pain medication or adjustment of pain medication (but no plan) was noted for another seven patients.

### Pain treatment

**Fig. 2:**
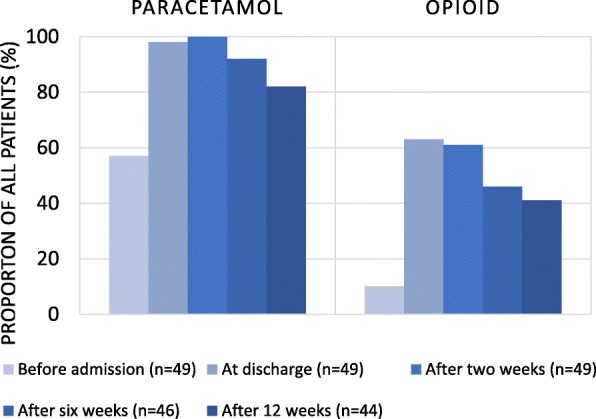
Proportion of patients with paracetamol and opioid prior to admission, at discharge from orthopaedic care and two, six and 12 weeks later

Table 1 shows the pain treatment distribution prior to and after discharge from orthopaedic care. Prior to admission, five patients were prescribed opioids for regular use, of which four in a slow-release formula (morphine or oxycodone). At discharge, 25 patients (51%) were prescribed opioids for regular use, of which 23 in a slow-release formula (mainly morphine or oxycodone). In total, 31/49 (63%) were opioid users (regular and/or as-needed) at discharge (Fig. 2), whereas the proportion of new opioid users was 26/44 (59%). Eight patients were prescribed warfarin at discharge, of which five in combination with paracetamol for regular use or as-needed in a dosage exceeding two grams.

**Table 1:** Pain treatment for all 49 primary care patients prior to admission, at discharge from orthopaedic care as well as two, six and 12 weeks later

	Prior to admission (*n* = 49)	At discharge (*n* = 49)	Two weeks after discharge (*n* = 49)	Six weeks after discharge (*n* = 46)	12 weeks after discharge (*n* = 44)
No pain medication (%)	18 (37%)	0	0	3 (6%)	7 (16%)
Paracetamol as-needed^a^ (%)	8 (16%)	5 (10%)	6 (12%)	10 (22%)	9 (21%)
Paracetamol regular use (%)	20 (41%)	43 (88%)	43 (88%)	32 (70%)	27 (61%)
Opioids as-needed^b^ (%)	2 (4%)	21 (43%)	24 (49%)	18 (39%)	13 (30%)
Opioids regular use (%)	5 (10%)	25 (51%)	22 (45%)	13 (28%)	12 (27%)
Other pain medications^a^ (%)	8 (16%)	6 (12%)	8 (16%)	7 (15%)	5 (11%)

a. Paracetamol was noted as regular **or** as needed use (not both)

b. Opioid was noted as regular **and/or** as-needed use

c. Tramadol, amitryptiline, pregabalin, prednisolone, gabapentin, paracetamol with codeine

Two and 6 weeks after discharge, one patient was prescribed NSAID (ibuprofen or naproxen).

Twelve weeks after discharge, 18/44 patients (41%) were still prescribed opioids for regular and/or as-needed use (Fig. 2). Four out of eight patients with a hospital plan for gradual reduction at discharge were still opioid users, as compared to 14/36 patients without such a plan (*p* = 0.697). Out of the patients with a primary care pain medication follow-up plan, 5/16 were still opioid users as compared to 13/28 without a plan (*p* = 0.325).

Overall, a greater and statistically significant proportion of patients not lost to follow-up were prescribed opioids 12 weeks after discharge (18/44, 41%) as compared to prior to admission (5/44, 11%) (***p*** **= 0.001**). This was also noted for patients prescribed paracetamol 12 weeks after discharge (36/44, 82%) as compared to prior to admission (26/44, 59%) (***p*** **= 0.021**). Pain medication for all patients over time is shown in Fig. 2.

### Pain treatment in regard to cognitive status and residency

A significantly lower proportion of patients with dementia were prescribed opioids as-needed compared to patients without dementia (Table 2), also noted for nursing home residents (10/31 vs 11/18, ***p*** **= 0.049**) including regular use (12/31 vs 13/18, ***p*** **= 0.024**) at discharge compared to community-dwelling patients.

**Table 2:** Pain treatment in primary care patients with/without dementia prior to admission (*n* = 49), at discharge (*n* = 49) from orthopaedic care and 12 weeks (*n* = 44) later

	No pain medication	Paracetamol	Opioids as-needed	Opioids regular use
Patient characteristics				
**Dementia**				
Prior to admission (*n* = 15)	6	9	1	2
At discharge (*n* = 15)	0	15	2 * (13%)	5
At 12 weeks (*n* = 14)	2	12	1 ** (7%)	2
**No dementia**				
Prior to admission (*n* = 34)	12	19	1	3
At discharge (*n* = 34)	0	33	19 * (56%)	20
At 12 weeks (*n* = 30)	5	24	12 ** (40%)	10

*/** Share of patients with dementia vs no dementia prescribed opioids as needed at discharge (*p = 0.006) and at 12 weeks (**p **= 0.026). No significant differences were otherwise noted**

## Discussion

### Summary of main findings

In this study on pain treatment in elderly and its follow-up in primary care, many patients still used paracetamol and/or opioids 12 weeks after discharge and follow-up plans for pain medication in primary care were seen for only one third of the patients. At discharge, nursing home residents received less opioids, and patients with dementia less opioids as-needed.

### Comparison with other studies

The patients in our study were elderly, often suffered from dementia and/or lived in nursing homes. Such patients commonly use MDD, which is associated with fewer drug treatment changes [28]. Hence, the high proportion of patients still using pain medication at 12 weeks may be considered to possibly represent persistence of drug treatment without clear indication and risk of harmful polypharmacy rather than persistent pain. In a similar Swedish cohort of patients with hip fractures [33], 45% of new opioid users still used potent or less potent opioids 6 months later. In our study, 41% (18/44) used opioids at 12 weeks, in line with hip fracture patients in a Danish study [34]. In another study, considerably fewer new opioid users still used opioids at 6 months [35]. However, that study population differed from ours by having a considerably lower median age and focusing on tibial shaft fractures. Similar to our study, 61% were new opioid users in the study by Dabbagh et al. [33], also in line with the Danish study [34]. The proportion of patients still using opioids after 6–12 months [33, 34] indicates a rather small decrease over time, although older age was associated with earlier opioid discontinuation [33]. Nevertheless, persistent pain in elderly fracture patients has also been previously noted [22, 36]. However, although increased use of analgesics is expected initially post-operatively, according to guidelines, opioid treatment should be reduced and ended, if pain remains as long as more than 3 months [7]. If needed, the patient should be referred to multidisciplinary treatment [7], as chronic pain requires a holistic rehabilitative approach rather than solely focusing on drug treatment [17]. In Finland, the introduction of an Acute Pain Service Out-Patient Clinic to bridge the gap between acute and chronic pain management has shown promising results by reducing acute pain medications and introducing other medications or non-pharmacological treatments when needed [37].

At 12 weeks, many patients (82%) in our study still used paracetamol, the first-line analgesic in nociceptive pain [2]. However, safety issues of long-term use have been raised [38], and continued use without clear indication should be avoided. The interaction of warfarin and paracetamol should also be taken into account, and follow-up such as more frequent monitoring of the INR (the International Normalized Ratio; a method used to monitor the effects of oral anticoagulants) planned [39].

Numerically fewer patients with (5/16) than without (13/28) a primary care follow-up plan used opioids at 12 weeks. Lack of follow-up may contribute to continued treatment without clear indication, with risk of side effects, polypharmacy and unnecessary costs. However, patients without any follow-up or gradual reduction plans also reduced their pain medication use. Whether this was initiated by the patients, the nurses or the GPs without noting it in the medical record is not known. It is possible these patients are actually better monitored by the GPs and the nursing staff than community-dwelling patients without medication aid and treatment therefore terminated when no longer needed. However, regular pain assessments as well as monitoring effect and side effects is a prerequisite for adequate pain treatment in elderly*.* If not routinely carried out, breakthrough pain, function and pain tolerance cannot be evaluated which may also lead to undertreatment.

Cognitively impaired elderly hip fracture patients receive significantly less opioids postoperatively [22, 40]. In our study, patients with dementia were prescribed opioids as needed to a significantly lower degree, possibly due to the difficulty with as-needed drugs for these patients. As Tracy et al. point out, pain treatment of patients with cognitive impairment needs special considerations, such as using scheduled dosing instead of as-needed, due to their inability to express their needs [3].

As for nursing home residents, poorer quality of drug treatment is commonly noted [24], and many suffer from untreated pain [20]. In our study, a significantly lower proportion of nursing home residents were prescribed opioids as compared to community-dwelling patients. Nursing home residents are possibly more fragile both physically and mentally, which may affect the perceived risk of opioid side effects and hence prescribing.

Both Japan and Sweden have a high life expectancy [41], and the proportion of elderly receiving long-term care (LTC) are similar [42]. The rapidly increasing aging population will increase the need for and costs due to LTC in both countries, although expected to increase more in Japan [42]. In Japan, a national LTC insurance plan was introduced for the elderly 20 years ago to provide the LTC and services according to need based on individual assessment of functional limitations. A professional care manager helps the person plan the care according to the individually established cost while the remainder is covered privately [42]. LTC covers home care, respite care and institutional care including nursing homes [43]. In Sweden, the municipality is obliged to provide the assistance needed, based on the decision of a municipal needs assessor [42]. This originates from the Ädelreform in 1992; an extensive structural change in the health field in which the municipalities took over the overall responsibility for healthcare and LTC in nursing homes or home care for community dwelling elderly [44]. The goal of the Ädelreform was to provide the municipalities with the necessary prerequisites to supply freedom of choice and security in the care of elderly [44].

As to the influence of genes and ethnicity, one study noted a lower metabolizing capacity of paracetamol for eastern Asians as compared to western Europeans, suggesting that they may be more susceptible to paracetamol induced toxicity [45]. Indeed, paracetamol was not approved for use in recommended doses in Japan until 2011, hence its use was low due to insufficient analgesic effect [46]. Even after the approval of standard dose, paracetamol is less often used than NSAID and often in low doses [46]. Prescription data from 2017 showed that half of new prescriptions of analgesics were NSAIDs while only about 10 % were paracetamol [46]. A traditional Japanese herbal medicine was also commonly prescribed, unlike in Sweden. Most patients were only prescribed one drug, and opioids were rarely prescribed [46].

In Sweden, prescription pattern data of analgesics during 2006–2015 showed a stable prevalence of opioids while an increase in paracetamol and decrease in NSAID (mainly for the elderly) prescribing was noted [47]. Paracetamol and opioids prescriptions increased with an increasing age and was most prevalent for patient aged 85 years or older [47].

### Implications for clinical practice and future research

Our results are well in-line with previous research, but adds the perspective of how it works in primary care, and may initiate a debate on how to improve the follow-up of these patients in primary care, for example with regular pain assessment.

However, the role played by the lack of pain medication follow-up plans is not yet entirely clear and should be further examined.

### Strengths and limitations

Pain management in elderly is a common and challenging task in primary care. The main strength was precisely the primary care perspective; as this is where these patients are mostly cared for.

The main weakness was the small study sample, due to difficulties with collecting medical case histories since the research group didn’t personally perform this task. Unfortunately, no better means of data collection was available. However, no subsequent bias was expected or noted. Further description of the possible loss in study population was not possible since all patients discharged from orthopaedic care could not be completely identified. Primary care research is challenging, and lacks experienced research nurses and the like for data collection. Yet, this makes primary care research all the more important, to describe for example how follow-up works in reality.

In addition, the results are derived from a limited number of GPs and their actions. Further, the medication information was partially based on reports from nurses in municipality care, assumed to be complete, but not always possible to double-check. Both patients with medication aid and nursing home residents were included, to obtain a study population of elderly patients quite possibly at the highest risk of drug-related problems and in great need of careful follow-up. Also, actual medication use is high for these patients, which strengthens the validity of the data. While forming a mixed sample, it also enables a description of two similar but distinct patient groups. Although the generalisability of our results to a different setting is uncertain, the accordance with previous research suggests a certain degree of credibility.

Also, doses of medications were not included, and decreases in daily doses may have been made without discontinuation of the treatment. We also lacked patient pain assessment and hence the degree of pain at each point is not known, which would have been helpful to clarify whether persistent treatment was indicated or a result of poor follow-up. However, this study describes a real situation where potentially risky treatment continues possibly due to lack of regular follow-up including pain assessment [16].

## Conclusion

Persistent pain medication was commonly noted, including opioid use at a point in time where it should be gradually reduced and ended, which may reflect poor follow-up of these vulnerable patients.

## Data Availability

The datasets generated and analysed during the current study are not publicly available since this was not included in the application for the ethics approval but are available from the corresponding author on reasonable request.

## References

[1] National Board of Health and Welfare. Indicators for good medication in the elderly. 2017.

[2] Arnstein P (2010). Balancing analgesic efficacy with safety concerns in the older patient. Pain Manag Nurs.

[3] Tracy B, Sean MR (2013). Pain management in older adults. Clin Ther.

[4] Falzone E, Hoffmann C, Keita H (2013). Postoperative analgesia in elderly patients. Drugs Aging.

[5] Barber JB, Gibson SJ (2009). Treatment of chronic non-malignant pain in the elderly: safety considerations. Drug Saf.

[6] Pergolizzi J, Boger RH, Budd K, Dahan A, Erdine S, Hans G (2008). Opioids and the management of chronic severe pain in the elderly: consensus statement of an International Expert Panel with focus on the six clinically most often used World Health Organization Step III opioids (buprenorphine, fentanyl, hydromorphone, methadone, morphine, oxycodone). Pain Pract.

[7] Medical Council in Skåne Region. Background material to local drug guidelines 2019.

[8] Swedish medical products Agency. Drug treatment of chronic pain in children and adults -treatment guidelines. Information from the Swedish medical products agency. 2017(3).

[9] World Health Organization Model List of Essential Medicines, 21st List. 2019. Contract No.: Licence: CC BY-NC-SA 3.0 IGO.

[10] Roberts E, Delgado Nunes V, Buckner S, Latchem S, Constanti M, Miller P (2016). Paracetamol: not as safe as we thought? A systematic literature review of observational studies. Ann Rheum Dis.

[11] Machado GC, Maher CG, Ferreira PH, Pinheiro MB, Lin CW, Day RO (2015). Efficacy and safety of paracetamol for spinal pain and osteoarthritis: systematic review and meta-analysis of randomised placebo controlled trials. BMJ.

[12] Chau DL, Walker V, Pai L, Cho LM (2008). Opiates and elderly: use and side effects. Clin Interv Aging.

[13] Guerriero F (2017). Guidance on opioids prescribing for the management of persistent non-cancer pain in older adults. World J Clin Cases.

[14] Hatahira H, Hasegawa S, Sasaoka S, Kato Y, Abe J, Motooka Y (2018). Analysis of fall-related adverse events among older adults using the Japanese Adverse Drug Event Report (JADER) database. J Pharm Health Care Sci.

[15] Webster LR (2017). Risk Factors for Opioid-Use Disorder and Overdose. Anesth Analg.

[16] Horgas AL (2017). Pain Assessment in Older Adults. Nurs Clin North Am.

[17] Smith BH, Hopton JL, Chambers WA (1999). Chronic pain in primary care. Fam Pract.

[18] Beijer HJ, de Blaey CJ (2002). Hospitalisations caused by adverse drug reactions (ADR): a meta-analysis of observational studies. Pharm World Sci.

[19] Landi F, Onder G, Cesari M, Gambassi G, Steel K, Russo A (2001). Pain management in frail, community-living elderly patients. Arch Intern Med.

[20] Hemmingsson ES, Gustafsson M, Isaksson U, Karlsson S, Gustafson Y, Sandman PO (2018). Prevalence of pain and pharmacological pain treatment among old people in nursing homes in 2007 and 2013. Eur J Clin Pharmacol.

[21] Tosounidis TH, Sheikh H, Stone MH, Giannoudis PV (2015). Pain relief management following proximal femoral fractures: Options, issues and controversies. Injury.

[22] Morrison RS, Siu AL (2000). A comparison of pain and its treatment in advanced dementia and cognitively intact patients with hip fracture. J Pain Symptom Manag.

[23] Jensen-Dahm CPH, Gasse C, Dahl JB, Waldemar G (2016). Postoperatve Treatment of pain after hip fracture in elderly patients with dementia. Dement Geriatr Cogn Disord.

[24] Bergman A, Olsson J, Carlsten A, Waern M, Fastbom J (2007). Evaluation of the quality of drug therapy among elderly patients in nursing homes. Scand J Prim Health Care.

[25] Johnell K, Fastbom J (2008). Multi-dose drug dispensing and inappropriate drug use: A nationwide register-based study of over 700,000 elderly. Scand J Prim Health Care.

[26] Wallerstedt SM, Fastbom J, Johnell K, Sjoberg C, Landahl S, Sundstrom A (2013). Drug treatment in older people before and after the transition to a multi-dose drug dispensing system--a longitudinal analysis. PLoS One.

[27] Sjoberg C, Edward C, Fastbom J, Johnell K, Landahl S, Narbro K (2011). Association between multi-dose drug dispensing and quality of drug treatment--a register-based study. PLoS One.

[28] Sjoberg C, Ohlsson H, Wallerstedt SM (2012). Association between multi-dose drug dispensing and drug treatment changes. Eur J Clin Pharmacol.

[29] Belfrage B, Koldestam A, Sjoberg C, Wallerstedt SM (2014). Prevalence of suboptimal drug treatment in patients with and without multidose drug dispensing--a cross-sectional study. Eur J Clin Pharmacol.

[30] Statistics Sweden. http://www.scb.se. Webpage first. Accessed 21 Dec 2016.

[31] National Board of Health and Welfare. Open comparisons 2018 -Care and nursing of elderly. Comparisons between municipalities and counties. 2018 [Available from: https://www.socialstyrelsen.se/globalassets/sharepoint-dokument/artikelkatalog/oppna-jamforelser/2019-2-2.pdf.

[32] IBM SPSS Statistics Data Editor 24 ed 2016.

[33] Al Dabbagh Z, Jansson KA, Stiller CO, Montgomery S, Weiss RJ (2016). Long-term pattern of opioid prescriptions after femoral shaft fractures. Acta Anaesthesiol Scand.

[34] Lindestrand AG, Christiansen ML, Jantzen C, van der Mark S, Andersen SE (2015). Opioids in hip fracture patients: an analysis of mortality and post hospital opioid use. Injury.

[35] Al Dabbagh Z, Jansson KA, Stiller CO, Montgomery S, Weiss RJ (2014). No signs of dose escalations of potent opioids prescribed after tibial shaft fractures: a study of Swedish National Registries. BMC Anesthesiol.

[36] Dasch B, Endres HG, Maier C, Lungenhausen M, Smektala R, Trampisch HJ (2008). Fracture-related hip pain in elderly patients with proximal femoral fracture after discharge from stationary treatment. Eur J Pain.

[37] Tiippana E, Hamunen K, Heiskanen T, Nieminen T, Kalso E, Kontinen VK (2016). New approach for treatment of prolonged postoperative pain: APS Out-Patient Clinic. Scand J Pain.

[38] McCrae JC, Morrison EE, IM MI, Dear JW, Webb DJ. Long-term adverse effects of paracetamol - a review. Br J Clin Pharmacol. 2018;84(10):2218–30. 10.1111/bcp.13656 PMC6138494 29863746

[39] Pinson GM, Beall JW, Kyle JA (2013). A review of warfarin dosing with concurrent acetaminophen therapy. J Pharm Pract.

[40] Spitz A, Moore AA, Papaleontiou M, Granieri E, Turner BJ, Reid MC (2011). Primary care providers’ perspective on prescribing opioids to older adults with chronic non-cancer pain: a qualitative study. BMC Geriatr.

[41] World Health Organization, Life expectancy at birth. http://gamapserver.who.int/mapLibrary/Files/Maps/Global_LifeExpectancy_bothsexes_2016.png. Accessed 18 Mar 2020.

[42] Lagergren M, Kurube N, Saito Y (2018). Future Costs of Long-term Care in Japan and Sweden. Int J Health Serv.

[43] Matsuda S (2002). The health and social system for the aged in Japan. Aging Clin Exp Res.

[44] The Swedish Riksdag. Changes in responsibilities in elderly care. 1990. Report No.: 1990/91:SoU9.

[45] Zurlinden TJ, Reisfeld B (2017). Characterizing the Effects of Race/Ethnicity on Acetaminophen Pharmacokinetics Using Physiologically Based Pharmacokinetic Modeling. Eur J Drug Metab Pharmacokinet.

[46] Ushida T, Matsui D, Inoue T, Yokoyama M, Takatsuna H, Matsumoto T (2019). Recent prescription status of oral analgesics in Japan in real-world clinical settings: retrospective study using a large-scale prescription database. Expert Opin Pharmacother.

[47] Backryd E (2018). Gender differences in dispensed analgesics in Sweden during 2006–2015 - an observational, nationwide, whole-population study. Int J Womens Health.

